# Alginate-*g*-PNIPAM-Based Thermo/Shear-Responsive Injectable Hydrogels: Tailoring the Rheological Properties by Adjusting the LCST of the Grafting Chains

**DOI:** 10.3390/ijms22083824

**Published:** 2021-04-07

**Authors:** Konstantinos Safakas, Sofia-Falia Saravanou, Zacharoula Iatridi, Constantinos Tsitsilianis

**Affiliations:** Department of Chemical Engineering, University of Patras, 26500 Patras, Greece; kostassaf@hotmail.com (K.S.); faliasaravanou@hotmail.com (S.-F.S.); iatridi@upatras.gr (Z.I.)

**Keywords:** alginate, PNIPAM-based graft copolymers, hydrogel, thermo-responsive, shear-responsive, LCST adjustment, sol–gel transition, rheological properties, injectability

## Abstract

Graft copolymers of alginate backbone and *N*-isopropylacrylamide/*N*-tert-butylacrylamide random copolymer, P(NIPAM_x_-*co*-NtBAM_y_), side chains (stickers) with various NtBAM content were designed and explored in aqueous media. Self-assembling thermoresponsive hydrogels are formed upon heating, in all cases, through the hydrophobic association of the P(NIPAM_x_-*co*-NtBAM_y_) sticky pendant chains. The rheological properties of the formulations depend remarkably on the NtBAM hydrophobic content, which regulates the lower critical solution temperature (LCST) and, in turn, the stickers’ thermo-responsiveness. The gelation point, *T_gel_*, was shifted to lower temperatures from 38 to 20 °C by enriching the PNIPAM chains with 20 mol % NtBAM, shifting accordingly to the gelation temperature window. The consequences of the *T_gel_* shift to the hydrogels’ rheological properties are significant at room and body temperature. For instance, at 37 °C, the storage modulus increases about two orders of magnitude and the terminal relaxation time increase about 10 orders of magnitude by enriching the stickers with 20 mol % hydrophobic moieties. Two main thermo-induced behaviors were revealed, characterized by a sol–gel and a weak gel–stiff gel transition for the copolymer with stickers of low (0.6 mol %) and high (14, 20 mol %) NtBAM content, respectively. The first type of hydrogels is easily injectable, while for the second one, the injectability is provided by shear-thinning effects. The influence of the type of media (phosphate buffer (PB), phosphate-buffered saline (PBS), Dulbecco’s modified Eagle’s medium (DMEM)) on the hydrogel properties was also explored and discussed. The 4 wt % NaALG-g-P(NIPAM_80_-*co*-NtBAM_20_)/DMEM formulation showed excellent shear-induced injectability at room temperature and instantaneous thermo-induced gel stiffening at body temperature, rendering it a good candidate for cell transplantation potential applications.

## 1. Introduction

In the last decades, hydrogels based on polysaccharide-biosourced natural macromolecules have attracted much interest due to their inherent biocompatibility, biodegradability, and nontoxicity, making them suitable candidates for applications in biomedicine and other healthcare applications [1,2,3,4,5]. Thanks to the pendant functional groups that they bear in their monomer units, polysaccharides can be easily modified by effortless reactions that enable them to form reversible three-dimensional (3D) networks through various non-covalent interactions. A commonly used modification is the grafting-to reaction with end-functional short polymeric chains that transforms them into associative graft copolymers [6].

Among others, alginate-based graft copolymers [7] have been designed to form 3D networks through the association of the pendant grafting chains, responding to a trigger like a temperature. In this case, poly(*N*-isopropylacrylamide), PNIPAM, was mainly used as the type of grafting chain due to its appropriate lower critical solution temperature (LCST), appearing at about 32 °C, below the physiological temperature [8,9,10,11,12,13,14,15,16]. Above a percolation concentration of alginate-g-PNIPAM (gelator) in water, the graft copolymer forms a 3D network upon heating, above a critical temperature, *T_gel_*, exhibiting a sol-to-gel transition. The rheological properties of the as-formed hydrogel and, in turn, its suitability for specific applications, depending on a number of factors, namely gelator concentration, grafting density, nature and length of grafts, and on the environmental conditions like temperature, pH, salinity and other solutes. To fulfill the demands for specific applications, all these factors must be regulated towards targeting tailor-made hydrogel properties, including injectability.

The regulation of *T_gel_* is crucial, provided that it determines the rheological properties of the hydrogel at room and physiological temperature. If it is very close to 37 °C, then the elastic modulus might be low, and the strength of the physical crosslinks might be weak, resulting in a viscoelastic response characterized by low relaxation times and relatively low viscosities, which is not desired for several applications, e.g., immobilization of stem cells. A favorable strategy to control *T_gel_*, keeping all the other factors constant, suggests the regulation of the LCST and/or the cloud point of the grafting chains [17,18,19,20]. It has been reported that the incorporation of hydrophobic or hydrophilic monomers in the LCST polymers in a random copolymer topology decreases or increases the LCST, respectively [18,21].

In a recent paper, we reported the properties of a hydrogel, based on a sodium alginate backbone, highly grafted by a thermo-responsive random copolymer of *N*-isopropylacrylamide, incorporating 10% mol of the hydrophobic *N*-*tert*-butylacrylamide, NaALG-g-P(NIPAM_90_-*co*-NtBAM_10_) [22]. This hydrogel system exhibited a *T_gel_* (determined at tan*δ* = 1) at about 32 °C, for *C_p_* = 13 wt % (at a fixed pH 7.4 and 0.2 M salinity). At room temperature, it behaves as a viscous liquid with low shear viscosity (three orders of magnitude higher than that of water), while above *T_gel_*, it forms a viscoelastic liquid, evolving to a gel at 37 °C, with an elastic modulus of the order of 100 Pa. More importantly, the system exhibits a shear/thermo-induced injectability and self-healing ability, as it responds instantly to sudden changes of shear and temperature. Nevertheless, all these properties may further be tuned, depending on the targeting application. Another issue is the gelator concentration, which is correlated with the mesh size of the network and, in turn, with its capacity to incorporate payloads, especially for cell transplantation. Thus, the gelator concentration should be as low as possible.

As demonstrated, the rheological properties of the system depend strongly on the hydrophobic strength of the P(NIPAM_90_-*co*-NtBAM_10_) grafting chains, which were reinforced by the presence of the NtBAM monomer [22]. It is known that the relative hydrophobicity of PNIPAM is low above its LCST, as PNIPAM never becomes totally hydrophobic, even well above its cloud point [23]. Thus, by using random copolymers enriched with hydrophobic moieties, we can regulate not only the LCST but also the hydrophobic association in terms of exchange dynamics of the stickers of the network that control the rheological properties [22,24,25,26,27]. The objective of the present work is to address the above issue by studying the effect of the hydrophobic content of the associative grafting P(NIPAM_x_-*co*-NtBAM_y_) chains of alginate-based graft copolymers, attempting to tailor the *T_gel_*, and in turns all the properties of the hydrogel formulations, including injectability at relatively low gelator concentration. This study ends up with a NaALG-g-P(NIPAM_80_-*co*-NtBAM_20_)/DMEM formulation, exhibiting excellent shear-induced injectability and instantaneous thermo-induced gel stiffening at physiological temperature, which can be a promising candidate for cell transplantation potential applications.

## 2. Results and Discussion

### 2.1. Synthesis and Characterization of Graft Copolymers

In this research study, a series of amino-functionalized P(NIPAM_x_-*co*-NtBAM_y_)-NH_2_ random copolymers with comparable molecular weight and different NtBAM content as well as a PNIPAM-NH_2_ homopolymer, were synthesized through conventional free radical polymerization (FRP) (synthesis details are presented in the Supplementary Materials). These polymers were characterized by potentiometric titration and proton nuclear magnetic resonance (^1^H NMR). From the acid–base titration of the polymers aqueous solutions, the number average molecular weight (M_n_) was determined, while from ^1^H NMR, the monomer molar composition, NIPAM/NtBAM, of the copolymers was calculated (more details in Figures S1–S4 in the Supplementary Materials). Table 1 summarizes the molecular characterization of the grafting chains. From the results on the molecular composition of the P(NIPAM_x_-*co*-NtBAM_y_)-NH_2_ copolymers, it can be seen that the NIPAM/NtBAM monomers molar ratio is in good accordance with the feed monomer composition throughout the synthesis of the copolymers.

At a second step, the aforementioned polymers were grafted onto an alginate backbone in aqueous media by forming an amide bond between the -NH_2_ groups of the amine-functionalized P(NIPAM_x_-*co*-NtBAM_y_) copolymers and the carboxylate groups of alginate, with the aid of EDC [11,22]. The alginate-based graft copolymers were characterized by ^1^H NMR (see details in Figures S5–S6 in the Supplementary Materials). The weight composition (NaALG/side chains, wt/wt) and the grafting density (grafting chains/NaALG, mol/mol) of the graft copolymers are displayed in Table 2.

**Figure 1:**
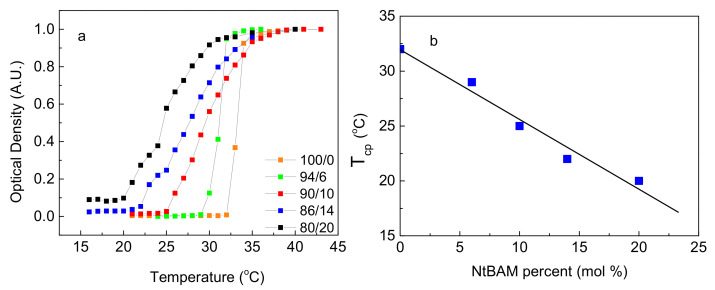
(**a**) Temperature dependence of the normalized optical density at 500 nm of 0.5% *w/v* aqueous solutions of ΡΝΙΡΑΜ-ΝΗ_2_ (orange), Ρ(ΝΙΡΑΜ_94_-*co*-NtBAM_6_)-ΝΗ_2_ (green), Ρ(ΝΙΡΑΜ_90_-*co*-NtBAM_10_)-ΝΗ_2_ (red) [22], Ρ(ΝΙΡΑΜ_86_*-co*-NtBAM_14_)-ΝΗ_2_ (blue), Ρ(ΝΙΡΑΜ_80_-*co*-NtBAM_20_)-ΝΗ_2_ (black). (**b**) *T_cp_* versus NtBAM content (mol %), the line is the linear fitting of the data (R^2^ = 0.962).

A UV-vis spectrophotometer was utilized to investigate the thermo-responsiveness of the grafting chains since PNIPAM is a well-known thermo-sensitive homopolymer with characteristic LCST behavior (at about 32 °C in water) [28,29]. The turbidimetry method was used to explore the thermo-responsiveness of all the synthesized grafting chains. In Figure 1a, the variation of the optical density at 500 nm at the temperature range 15–45 °C of aqueous solutions (0.5% *w/v*) of the P(NIPAM_x_-*co*-NtBAM_y_) random copolymers, including the PNIPAM homopolymer, is presented.

As can be seen, the optical density increases abruptly above a certain temperature for the ΡΝΙΡΑΜ-ΝΗ_2_ homopolymer and the Ρ(ΝΙΡΑΜ_94_-*co*-NtBAM_6_)-ΝΗ_2_ copolymer. However, for the three copolymers with the higher NtBAM content, Ρ(ΝΙΡΑΜ_90_-*co*-NtBAM_10_)-ΝΗ_2_, Ρ(ΝΙΡΑΜ_86_-*co*-NtBAM_14_)-ΝΗ_2_ and Ρ(ΝΙΡΑΜ_80_-*co*-NtBAM_20_)-ΝΗ_2_, the optical density increases gradually. This is likely due to the heterogeneity of the monomer composition in the copolymers, arisen from the conventional free radical polymerization method. The NtBAM content is an average value, and the sample is constituted of a mixture of statistical copolymers with NtBAM-richer and NIPAM-richer rather than the average value compositions [22,30]. The cloud point temperature (*T_cp_*) was defined as the temperature where the optical density of the studied polymer solution increases promptly. The *T_cp_* values of the copolymers are included in Table 1. Figure 1b depicts a decreasing linear dependence of *T_cp_* as a function of the NtBAM hydrophobic monomer content, as expected [31]. This behavior allows fine *T_cp_*-tuning by adjusting the molar content of the hydrophobic comonomer during copolymerization with ΝΙΡΑΜ.

### 2.2. Thermo-Induced Gelation of Graft Copolymers

**Figure 2:**
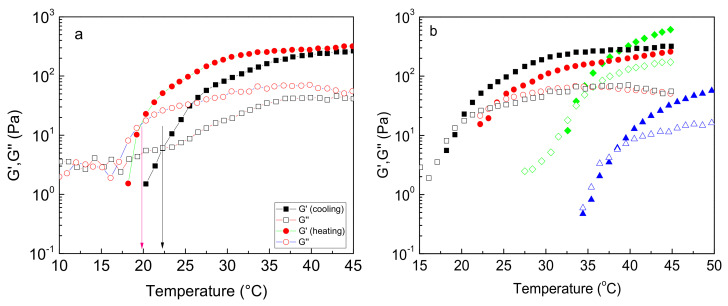
(**a**) Temperature dependence of *G’* (closed), *G”* (open) at 1 Hz and strain amplitude of 0.1% for the NaALG-*g*-P(NIPAM_80_-*co*-NtBAM_20_) graft copolymer in water (*C_p_* = 5 wt %). The different symbols indicate heating (red) and cooling (black) ramp both at a rate of 1 °C/min. The arrows indicate the temperature at *G’= G”*. (**b**) *G’* (closed), *G”* (open) versus temperature (heating ramp) at the same conditions and C_p_ for different graft copolymers: NaALG-*g*-ΡΝΙΡΑΜ (triangles, blue), NaALG-*g*-Ρ(ΝΙΡΑΜ_94_-*co*-NtBAM_6_) (diamonds, green), NaALG-*g*-Ρ(ΝΙΡΑΜ_86_-*co*-NtBAM_14_) (circles, red), NaALG-*g*-Ρ(ΝΙΡΑΜ_80_-*co*-NtBAM_20_) (squares, black).

As we have already reported, graft copolymers of NaALG, grafted with PNIPAM-based thermoresponsive chains, form 3D percolated networks upon heating, exhibiting a sol-to-gel transition. To explore the influence of the hydrophobic NtBAM content of the PNIPAM-rich grafting chains on the thermo-induced rheological properties of the alginate-based graft copolymer hydrogels, oscillatory shear measurements were conducted. Keeping constant the polymer concentration, *C_p_*, at 5 wt % (above the percolation threshold), temperature sweeps were performed in the linear viscoelastic regime (constant strain amplitude of 0.1%) and at a fixed frequency of 1 Hz. First, a cooling procedure was conducted from high to low temperatures, followed by a heating procedure with the same rate of 1 °C/min in all cases. Figure 2a demonstrates an example of this procedure concerning the NaALG-*g*-P(NIPAM_80_-*co*-NtBAM_20_) graft copolymer. As seen, at high temperatures, *G’* dominates to *G”*, manifesting a gel-like behavior. Upon decreasing temperature, both moduli start to decrease, crossing each other at a certain temperature, denoted as *T_gel_*. Below this temperature, the system behaves as a flowing liquid (sol) since the loss modulus prevails the storage one (*G” > G’*). Upon heating, the system follows a different pathway exhibiting hysteresis, which is more pronounced in the vicinity of the *G’’/G’* crossover point. The *T_gel_* (heating) appears about 2–3 °C lower than the *T_gel_* (cooling). This hysteresis tends to vanish as the NtBAM content decreases (Figure S7 in the Supplementary Materials). In the copolymer bearing pure PNIPAM side chains, the hysteresis seems to be negligible. The observed hysteresis depends on the ramp rate. As observed for the NaALG-*g*-PNIPAM, the difference between *T_gel_* (heating) and *T_gel_* (cooling) is practically zero at 0.5 °C/min (slow ramp) and reaches ~4 °C when a 10 °C /min, fast ramp, is applied (Figure S8 in the Supplementary Materials), revealing that it is a matter of equilibration, correlated with the dynamics of the network. This is corroborated by the fact that in the sol state, the system is in an equilibrium state (no network exists). This also appears at high temperatures (gel state), where sufficient time during heating allows equilibration again. In the following, we discuss only the heating procedure data with a heating rate of 1 °C/min. In Figure 2b, the results of all the polymer systems are displayed. The data are shifted to lower temperatures as the hydrophobic content (NtBAM moieties) of the thermo-responsive associative side chains increases.

**Figure 3:**
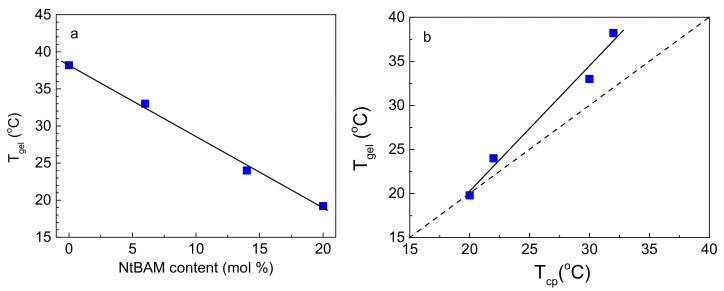
(**a**) T_gel_ as a function of NtBAM content (mol %) of the grafting chains; the straight line is a linear fit of the data (R^2^ = 0.993). (**b**) *T_gel_* of the graft copolymer versus *T_cp_* of the grafting chains. The dashed line denotes *T_gel_* = *T_cp,_* and the solid line is a linear fit of the data (R^2^ = 0.973).

In Figure 3a, *T_gel_* is plotted as a function of NtBAM content (mol %), exhibiting a linear decay with the hydrophobic enrichment of the grafts. It should be emphasized that although the grafting density and, in turn, the total NIPAM/NtBAM percentage of the copolymers differ, and despite their molecular polydispersity, *T_gel_* is very well correlated with the NtBAM content of the grafting chain (R^2^ = 0.993), showing that this is the determining factor. Any other correlation, e.g., *T_gel_* vs. NtBAM_total_ (mol %), fails. However, when the grafting density is remarkably high (see our previous work [22]), there is a deviation from this relationship. The above finding implies that *T_gel_* should also be correlated with *T_cp,_* which is controlled by the NtBAM content, as shown in Figure 1. Hence, the lower the LCST of the grafting chains, the lower the *T_gel_* should be. Indeed, by plotting *T_gel_* versus *T_cp_* (Figure 3b), a linear function can be observed, clearly showing their relationship. Another interesting observation arises from the comparison of the data with the line *T_gel_* = *T_cp_*. At high NtBAM content (20% mol), the two temperatures coincide, while an increasing deviation *T_gel_* > *T_cp_* appears as NtBAM content decreases to pure PNIPAM. This should be attributed to the strength of the side chains’ hydrophobic association, which depends both on temperature and NtBAM content. Note that PNIPAM is never entirely hydrophobic, even at sufficiently higher temperatures than its cloud point [23]. Thus, by enriching PNIPAM with NtBAM hydrophobic moieties, the side chains exhibit increasing stickiness, and the gel point is manifested closer to the phase transition (LCST type) of the grafting chains.

Here we should discuss *T_gel_*, as it is defined at the crossover temperature where G’ = G”, as usually reported in the literature. This is not a real first-order transition as at *T* > *T_gel_* close to T_gel_, the solution still flows, behaving as a viscoelastic liquid, and it becomes an opaque free-standing gel at temperatures well above the sol-to-gel transition. Thus, *T_gel_* denotes, in fact, the onset of thermo-induced gelation either by heating (LCST-type) or by cooling (UCST-type) [32]. Provided that *T_gel_*, determined by the temperature sweep experiments, depends on the applied frequency (verified with the NaALG-*g*-PNIPAM sample), it is referred to as apparent gelation temperature [5,32,33]. This is attributed to the dynamic character of association and deals with the lifetime of the physically formed reversible crosslinks concerning the experimental time. Thus, to evaluate whether real gelation occurs, frequency sweep measurements at various temperatures above *T_gel_* should be examined.

**Figure 4:**
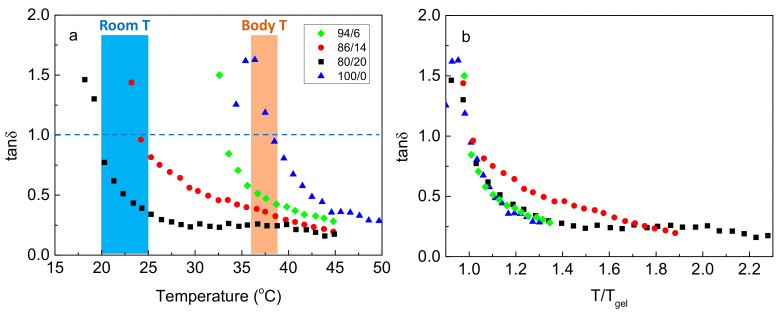
(**a**) Temperature dependence of the loss tangent (tan*δ*) at 1 Hz for the systems indicated. The two-colored zones mark the room and body temperature. (**b**) Loss tangent (tan*δ*) as a function of the normalized temperature *T/T_gel_* (symbols as in (**a**)).

The data of Figure 2b can also be demonstrated using a single parameter, namely loss tangent (tan*δ* = *G”/G’*), which is a measure of the viscoelasticity of the material (Figure 4). The temperature at tan*δ* = 1 denotes *T_gel_*, and as tan*δ* decreases below unity upon heating, the material’s elasticity increases, in expense to its viscous response. As observed, tan*δ* decreases steadily with temperature (Figure 4a) in all systems, tending to converge at high temperature, well above *T_gel_*. By plotting tan*δ* as a function of temperature normalized by *T_gel_* (Figure 4b), the data almost superimpose to a master curve (with small deviation for the 86/14 polymer), showing that the enrichment of the sticky side chains of the graft copolymer, simply shifts the gel window at lower temperatures and that the gelation mechanism, i.e., heat-induced hydrophobic association, is identical. Evidently, gelation is a gradual process and not a sharp sol-to-gel transition.

### 2.3. Rheological Properties at a Given Temperature and Responsiveness

**Figure 5:**
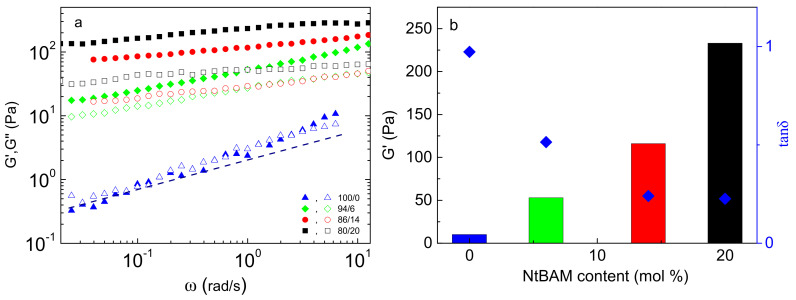
Frequency dependence of G’ (closed), G” (open) (**a**) for the NaALG-*g*-P(NIPAM_x_-*co*-NtBAM_y_)/water systems of different monomer compositions in the grafting chains, as indicated, at 37 °C. The slope of the dashed line is 0.5. (**b**) Storage modulus, *G’* (at 1 rad/s) (bars) and tan*δ* (diamonds, right axis) versus NtBAM content (mol %) of the grafting chains.

The comparison of the viscoelastic response of the different systems is useful to also be examined at specific temperature values. For instance, in biomedical applications, the interest focuses on the state of the formulations at room and body temperature (marked zones in Figure 4a). In Figure 5a, the frequency (ω) dependence of the moduli for all the systems at 37 °C is displayed. As can be observed, the shift of *T_gel_* at lower temperatures obviously affects the gelation state at 37 °C. The copolymer solution with pure PNIPAM side chains exhibits nearly liquid-like behavior as *G’* and *G”* almost coincide (tan*δ* close to unity), and both are dependent on frequency. The exponent of the power-law dependence of both moduli is slightly higher and close to 0.5, showing that the system at 37 °C is in the vicinity of the transition point between liquid-like and solid-like behavior [34]. As the side chains are enriched with the hydrophobic NtBAM moieties, the storage modulus dominates the loss modulus in the entire frequency range investigated, exhibiting solid-like behavior. The *G’* (Figure 5b) increases and the tan*δ* (Figure S9 in the Supplementary Materials) decreases with increasing the hydrophobic content of the side chains of the graft copolymers, clearly indicating the reinforcement of the polymer networks. Considering that the rubber elasticity theory is valid for the present network systems, the elastic modulus, *G_N_*, is proportional to the number density of the elastically active chains, *n* (intermolecular bridging), *G_N_ = nK_B_T*, where *K_B_*, *T* are the Boltzmann’s constant and the absolute temperature, respectively. Therefore, the increase of the storage modulus (obtained at 1 rad/s) at 37 °C, demonstrated in Figure 5b, suggests that the hydrophobic enrichment of the sticky side chains imposes a higher number of the network strands at a given temperature. Particularly, from the pure PNIPAM to 80/20 PNIPAM/NtBAM grafting chains, the storage modulus increases two orders of magnitude, while the loss tangent decreases from 0.97 to 0.22 (Figure 5b), suggesting remarkable improvement of the elasticity of the hydrogel at the physiological temperature.

**Figure 6:**
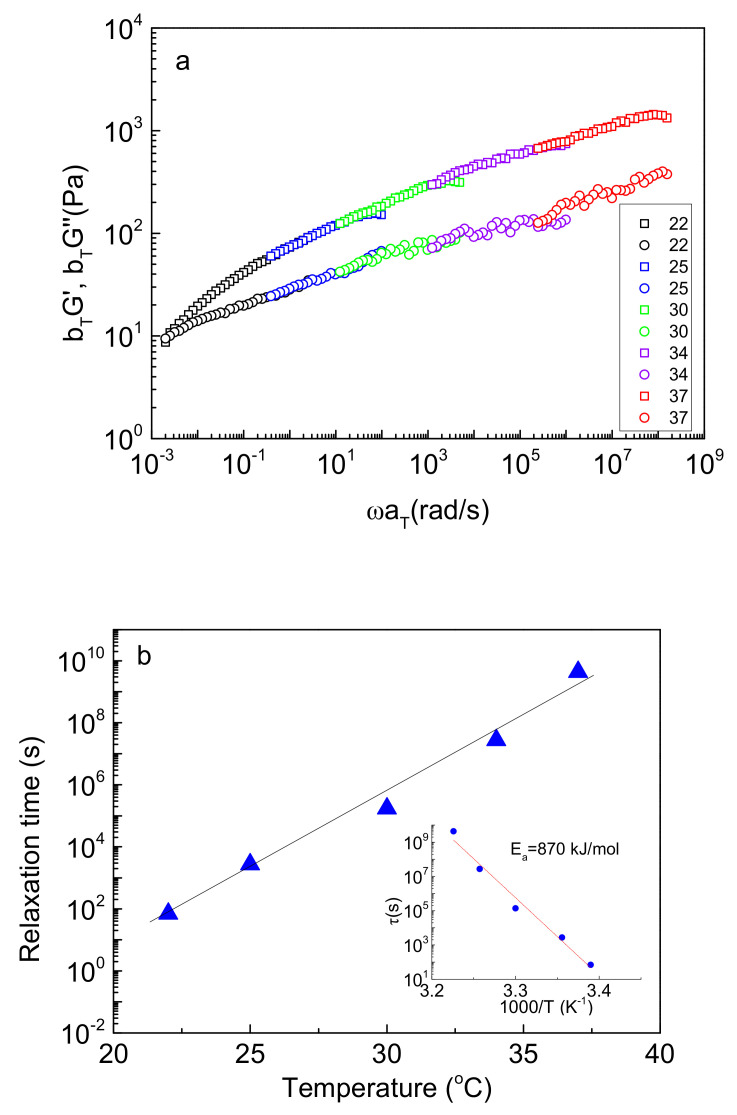
(**a**) Time–temperature superposition master curve of G’ (squares) and G” (circles) at the reference temperature of 22 °C and (**b**) temperature dependence of relaxation time, *τ* and the corresponding Arrhenius plot (inset) for the NaALG-*g*-P(NIPAM_80_-*co*-NtBAM_20_) graft copolymer/water system (*C_p_* = 5 wt %). The solid lines are linear fits of the data.

Furthermore, as seen in Figure 5a, the terminal relaxation zone is not visible for the NaALG-*g*-P(NIPAM-*co*-NtBAM) systems, implying long relaxation times. To evaluate them, frequency sweep experiments were accomplished in various temperatures from 22–37 °C for the 80/20 copolymer (Figure S10 in the Supplementary Materials) to apply the time–temperature superposition principle. Figure 6 demonstrates *b_T_G’* and *b_T_G”* versus the reduced frequency *ωa_T_*, where *a_T_*, *b_T_* is the horizontal and vertical shift factors, respectively, using 22 °C as the reference temperature. A satisfactory master curve was achieved for the NaALG-*g*-P(NIPAM_80_-*co*-NtBAM_20_) graft copolymer/water system, which, however, exhibits a peculiarity. The loss modulus, *G”*, continuous to increase above the crossover point. Such behavior has not been reported by well-defined ABA triblock copolymer hydrogelators with sticky ends, while similar behavior has been reported for entangled star-shaped branched polymers [35] or to our previous report that dealt with the highly branched NaALG-*g*-P(NIPAM_90_-*co*-NtBAM_10_) (C_p_ = 10 wt %) copolymer [22]. This trend may be attributed to the exponential retarded relaxation modes of the grafting chains [36].

The terminal relaxation time, *τ*, at the reference temperature, T = 22 °C, was determined, by the crossover frequency, *τ = 1/ω_c_*, at 70 s. By using the *a_T_* shift factors, *τ* can be evaluated at various temperatures through the equation 1 *τ = τ_ref_ a_T_*. Figure 6b demonstrates an exponential increase of *τ* with temperature. The relaxation time increases about eight orders of magnitude from 22 to 37 °C. More important, from pure PNIPAM to 80/20 PNIPAM/NtBAM grafting chains, *τ* increases from 0.1 to 4.3 × 10^9^ s, hence more than ten orders of magnitude, revealing a strong effect for just 20 mol % hydrophobic enrichment of the sticky grafting chains. Thus, at 37 °C, the NaALG-*g*-P(NIPAM_80_-*co*-NtBAM_20_)/water system behaves as a “frozen” hydrogel, resembling a permanent, chemically crosslinked gel.

In the inset of Figure 6b, the data were plotted to apply the Arrhenius Equation (1):

*τ = τ_o_ exp[−E_a_/RT]* (1)

where *E_a_* is the apparent activation energy, which represents the energy barrier the network stickers need to overcome to escape from their crosslinking nanodomains (micellar cores), allowing relaxation [24,32]. *E_a_* was estimated 870 kJ/mol, which is comparable with the 619 kJ/mol reported for a triblock copolymer constituted of poly(*N,N*-dimethylacrylamide), end-capped with PNIPAM-based random copolymers of poly(NIPAM_82_-*co*-butyl acrylate_18_) and poly(NIPAM_95_-*co*-butyl acrylate_5_) [37]. In this copolymer, the PNIPAM sticky end-blocks have been enriched with the hydrophobic butyl acrylate moieties in 82/18 (mol %) content. As it is known, *E_a_* is proportional to *N^2/3^γ*, where *N* is the degree of polymerization of the associative chains, while *γ* is their surface tension with the solvent, which, in turn, is related to the Flory–Huggins polymer/solvent interaction parameter *χ* [38]. Thus, the enrichment of PNIPAM with hydrophobic monomers increases *γ*, analogously affecting *E_a_*. Provided that in our case, *N* is higher (146 versus 89) and *γ* slightly higher (hydrophobic content: 20 mol % versus 18 mol %), the higher *E_a_* estimated herein seems fairly reasonable (see Supplementary Materials). Note that the different copolymer architecture (graft versus triblock) may also affect *E_a_* when the number of stickers per chain (grafting chains or end blocks) is considerably different since the network structure and connectivity affect the dynamics of the network as well [22,32].

The rheological properties of the hydrogel formulations at room temperature (20–25 °C) are also very important since they decisively influence their injectability. For the gelators with low hydrophobic content (e.g., 100/0, 94/6) in their stickers, the *T_gel_* is well above the room temperature (Figure 4a), and hence, they exhibit a sol behavior at room temperature being easily injectable. For the gelators with higher hydrophobic content, exhibiting *T_gel_* in the room temperature regime, the hydrogels show significant viscoelasticity, as can be seen in Figure S11 (see in the Supplementary Materials). Especially for the NaALG-*g*-P(NIPAM_80_-*co*-NtBAM_20_) graft copolymer, with the characteristics of *G’* = 81.7 Pa, tan*δ* = 0.366 (at 10 rad/s) and *τ* = 79 s, signifying a weak “gel” (viscoelastic fluid), injectability is not obvious.

**Figure 7:**
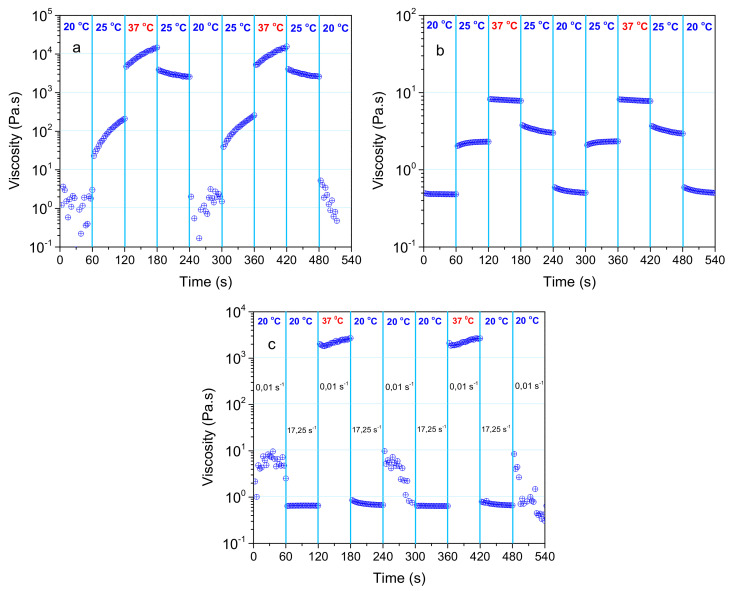
Shear viscosity versus time of a 5 wt% NaALG-*g*-P(NIPAM_80_-*co*-NtBAM_20_) aqueous solution subjected to stepwise temperature switch at a constant shear rate of 0.01 s^−1^ (**a**) and of 17.25 s^−1^ (**b**). In (**c**), the sample was subjected to consecutive conditions by simultaneously varying shear rate and temperature, e.g., from 0.01 s^−1^/ 20 °C to 17.25 s^−1^/ 20 °C to 0.01 s^−1^/ 37 °C in three cycles.

As reported recently, the injectability is correlated with the thermal and shear rate responsiveness of the formulation, as arisen from their thermo-sensitivity (Figure 2) and shear thinning behavior (Figure S12 in the Supplementary Materials), respectively [22]. Thus, consecutive shear viscosity time-sweep experiments were conducted at specific temperatures (20, 25, 37 °C) and under shear rates of 0.01 and 17.25 s^−1^. The first low value approaches the zero-shear viscosity (rest) and the second one exemplifies the shear rate applied through a 28-gauge syringe needle [39]. First, the hydrogel was subjected to consecutive stepwise temperature switch, under constant shear rates and at time intervals of 60 s. Figure 7a,b shows the shear viscosity changes upon temperature switch, responding instantaneously to the stepwise temperature variation. More important, the viscosity profiles are reproducible, either on increasing or decreasing temperature, demonstrating excellent temperature responsiveness.

Comparing the viscosity profiles at the same temperature but under different shear rates (0.01 s^−1/^Figure 7a versus 17.25 s^−1/^Figure 7b), the following effects can be observed.

First, the lower the shear rate, the higher the viscosity, at the same temperature, in agreement with the shear-thinning behavior of the formulation (Figure S12 in the Supplementary Materials). Second, a steady-state cannot be established under the low shear rate of 0.01 s^−1^ within the time interval investigated, while at the high shear rate of 17.25 s^−1^, a steady-state can be achieved. These phenomena should be attributed to the long relaxation times of the network that are higher than the experimental time, especially under low shear. At a high shear rate, the network is disrupted, and the increasing mobility of the macromolecules allows steady-state establishment. Finally, the viscosity profiles at the same temperature differ notably when the temperature changes from lower (increasing T) versus higher temperatures (decreasing *T*). This should be correlated with longer relaxation times at higher temperatures (Figure 6b).

In Figure 7c the experiment was designed to simulate conditions similar to those of injection through a 28 gauge needle syringe. The injection temperature was set at 20 °C since, at this temperature, the shear viscosity under the shear rate of 17.25 s^−1^ (Figure 7b) is lower than 1 Pa, which is an acceptable value for injection. As observed, the shear viscosity rises instantly more than three orders of magnitude when the conditions are switched from 17.25 s^−1^/ 20 °C to 0.01 s^−1^/ 37 °C. The viscosity profiles are fairly reproducible, irrespectively of the direction of temperature change (heating or cooling). This experiment confirms the excellent injectability of the system.

### 2.4. Rheological Properties in Various Media

**Figure 8:**
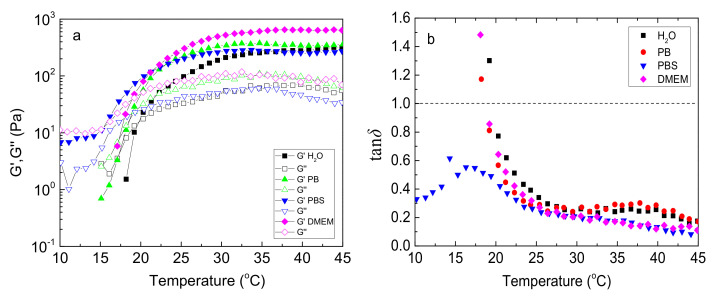
Temperature dependence of G’ (closed), G” (open) (**a**) and tanδ (**b**) (at 1 Hz, γ = 0.1%, heating rate 1 °C/min) for 5 wt % NaALG-*g*-P(NIPAM_80_-*co*-NtBAM_20_) graft copolymer in various media: water, phosphate buffer (PB), phosphate-buffered saline (PBS), Dulbecco’s modified Eagle’s medium (DMEM) (indicated in the inset).

In the next step, we examined the rheological behavior of the NaALG-*g*-P(NIPAM_80_-*co*-NtBAM_20_) graft copolymer in various media used for biological research and/or biomedicine applications, namely phosphate buffer (PB, 1 mM), phosphate-buffered saline (PBS, 1 mM, 0.135 M NaCl) and Dulbecco’s modified Eagle’s medium (DMEM). The same temperature sweep experiments were conducted, as described in Figure 2a, applying cooling/heating of the formulations from 45 to 10 to 45 °C with a rate of 1 °C/min (Figure S13 in the Supporting Information). In Figure 8, the temperature dependence of *G’*, *G”* moduli and tan*δ* are demonstrated for the heating sweep procedure. The general thermal behavior in all media was similar; that is, a remarkable increase of the network elasticity above a certain temperature, *T_gel_*, as manifested by the storage moduli rise and the loss tangent decrease upon heating. However, in PBS media, although the *G’*, *G”* augment promptly above 15 °C (Figure 8a), they never cross each other, regardless of cooling or heating procedure. G’ always prevails G” within the temperature range investigated, implying that the network is not entirely disrupted at low temperatures, even well below the *T_cp_* of the sticky grafting chains. More important, tan*δ* decreases again at low temperatures below 15 °C (Figure 8b), showing an increased elasticity and revealing the so-called cold gelling [14]. This effect should be attributed to the presence of relatively high salt content, and especially potassium cations, which promote the cold gelling through the association of the mannuronic sequences of the alginate backbone [14].

Furthermore, the presence of salts slightly affects *T_gel_* (Table S2 in the Supplementary Materials). This should be attributed to the shift of the *T_cp_* of the thermosensitive PNIPAM-rich pendant sticky grafting chains due to the well-known salting-out effect [20,40]. More pronounced effects were observed to the G’, G” moduli. Especially in DMEM media, the storage modulus is higher than the one in pure water, more than 240% at the physiological temperature, although *T_gel_* shifted to a lower value just 1 °C (Table S2 in the Supporting Information), meaning that the gelation window practically remains in the same temperature range. DMEM is a multi-constituent medium comprising salts, amino acids, vitamins, and glucose and is used for cell culture and transplantation. All these ingredients may affect the network structure and connectivity since they interact with the gelator through non-covalent interactions, either with the alginate backbone (polyelectrolyte screening) and/or PNIPAM-based grafting chains (salting-out effect). Provided that in salts, divalent cations like Ca^2+^ are also included, it is very likely that additional crosslinking occurs due to ionic interactions with the -COO^−^ anions of the alginate backbone, which could justify the G’ augmentation [39].

### 2.5. Injectable Hydrogel in Cell Culture Fluid Media (DMEM)

Cell transplantation through injection strategies into host tissues is one of the potential biomedical applications that have attracted much attention. It is known that the injection of cells within simple formulations through a needle significantly reduces their viability due to exposing the cells to substantial extensional flows that can damage cell membranes [41]. A plausible strategy to overcome this problem is to use as the cell carrier a two-step gelling system, exhibiting shear-thinning and self-healing properties [42,43]. Shear- and thermo-responsive hydrogels seem to be good candidates for this purpose since they form a weak gel at room temperature, protecting the cells, and a stiff gel after injection at a physiological temperature that can retain the cell at the target location. Here, we explore the response of the DMEM formulation to the injection requirements.

**Figure 9:**
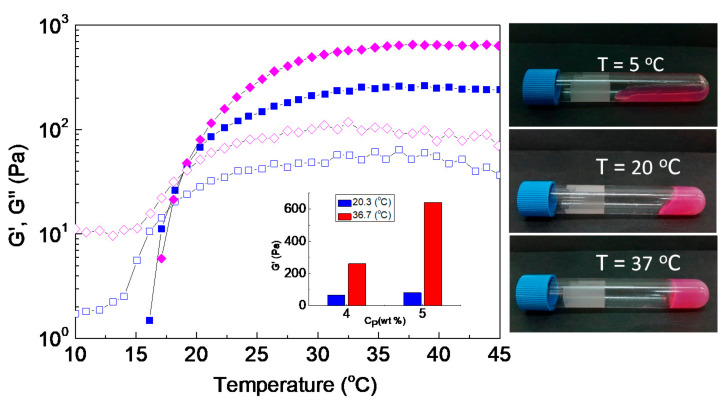
Temperature dependence of *G’* (closed), *G”* (open) (at 1 Hz, γ = 0.1%, heating rate 1 °C/min) for NaALG-*g*-P(NIPAM_80_-*co*-NtBAM_20_) graft copolymer in Dulbecco’s modified Eagle’s medium (DMEM) at 5 wt % (diamonds) and 4 wt % (squares) polymer concentration. The inset demonstrates the storage modulus, *G’*, at room and body temperatures. The photos (**right**) illustrate the solutions of 4 wt %, equilibrated at the indicated temperatures.

Figure 9 shows the heating sweep data for the NaALG-*g*-P(NIPAM_80_-*co*-NtBAM_20_)/DMEM formulations at 4 and 5 wt % polymer concentrations. The gelator concentration is another factor that can tune the hydrogel properties. From the data of Figure 9, it seems that concentration affects slightly *T_gel_*, about 1 °C (Table S2 in the Supplementary Materials), while it significantly affects the network elasticity well above *T_gel_*. For instance, at room temperature, *G’* is about similar for both 4 and 5 wt % concentrations. On the contrary, at body temperature, when C_p_ decreases from 5 to 4 wt %, *G’* also decreases (by a factor of 2.4) (inset of Figure 9). The formulation exhibits sol state at low temperature (e.g., at 5 °C), viscoelastic liquid (soft gel) at 20 °C and free-standing gel (stiff gel) at 37 °C (Figure 9, digital photos).

**Figure 10:**
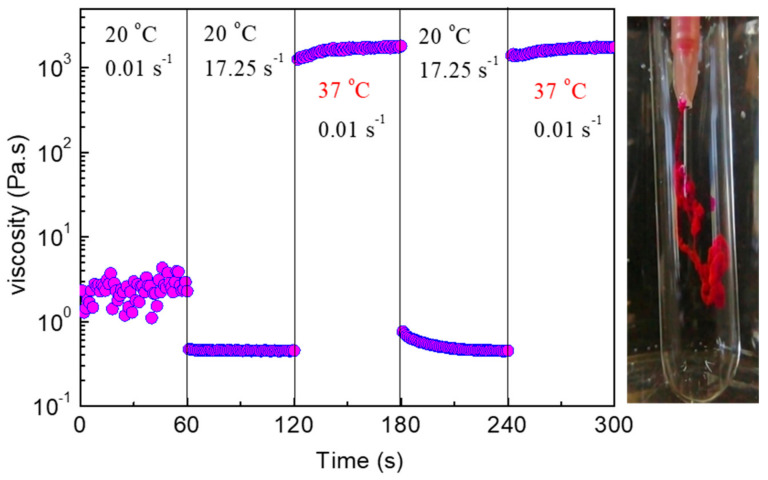
Shear viscosity versus time of a 4 wt % NaALG-*g*-P(NIPAM_80_-*co*-NtBAM_20_)/DMEM formulation subjected to consecutive variations of shear rate and/or temperature, as indicated, in time intervals of 60 s. The digital photo (**right**) shows injection of the formulation through a 28 g needle in water, regulated at 37 °C.

The enhanced values of moduli in DMEM media (C_p_ = 5 wt %) allow lowering of C_p_, which is beneficial, as we have already mentioned in the Introduction. Thus, focusing on the 4 wt % NaALG-*g*-P(NIPAM_80_-*co*-NtBAM_20_)/DMEM formulation, we examined its injectability by consecutive shear and oscillatory time sweep experiments. Figure 10 illustrates the shear viscosity experiment that comprises sudden changes of the shear rate/ temperature conditions, simulating an injection with a 28 g needle. The shear viscosity decreases under high shear at 20 °C, obtaining values lower than 1 Pa.s, while it rises instantaneously more than three orders of magnitude (6 orders of magnitude higher than the viscosity of the medium) at physiological temperature, forming a stiff gel. The results are quite reproducible, as can be observed by the second cycle of changes in the same experiment. These data imply excellent injectability, which was also verified by optical observation when injecting the formulation at room temperature in a water medium, regulated at 37 °C (see the digital photo in Figure 10).

**Figure 11:**
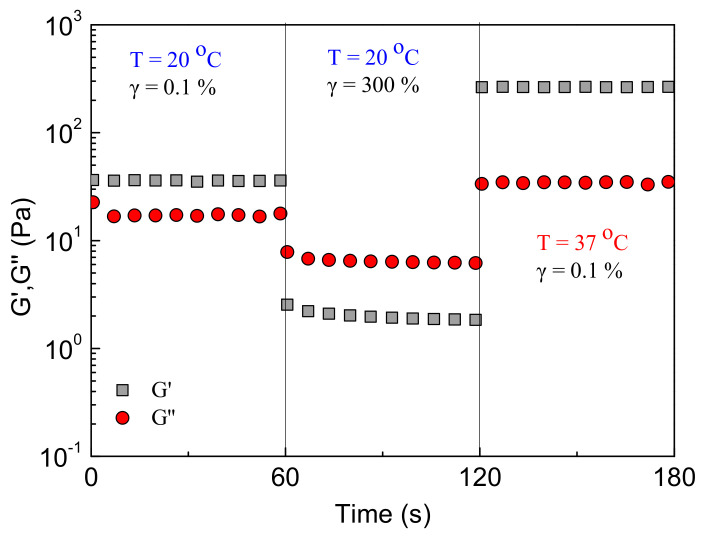
Time dependence of G’ (squares) and G” (circles) at 1 Hz, under different conditions of temperature and strain amplitude, as indicated, for the 4 wt % NaALG-*g*-P(NIPAM_80_-*co*-NtBAM_20_)/DMEM formulation.

An oscillatory shear experiment was designed to explore the formulation’ response to high shear deformation and gel recovery upon cessation of strain. The formulation was subjected to stepwise time sweep under different conditions: at 20 °C, applying a shear amplitude, *γ*, of 0.1% (linear viscoelastic regime); after 60 s, *γ* was switched to 300% (well beyond linear viscoelastic regime); after further 60 s, the temperature was increased at 37 °C and simultaneously *γ* was lowered back to 0.1%. The results are presented in Figure 11. As observed, at 20 °C and low strain, the formulation behaves as a weak gel (*G’ > G”*), with a storage modulus of 35 Pa and tan*δ* = 0.47. Upon applying high strain in the nonlinear regime, the network is disrupted instantly, and the formulation flows (*G’ < G”* and tan*δ* = 3.3) with low *G’* ~2 Pa. Finally, in the third step, a stiff gel is recovered at 37 °C (*G’ > G”*), as suggested by the high value of *G’* (260 Pa) and the low value of tan*δ* = 0.13.

Again, the system responds instantly to any variation of temperature and strain. Notably, the storage modulus instantaneously increases more than two orders of magnitude after network disruption, showing excellent self-healing ability as well (see also Figure S14 in the Supplementary Materials).

It is interesting to compare the thermomechanical response of the present system with the two-component material, namely SHIELD (shear-thinning hydrogel for injectable encapsulation and long-term delivery), constituted of star-shaped polyethylene glycol (8-armed PEG tethered with proline-rich peptides (P) and a PNIPAM chain) copolymer and an engineered recombinant protein (C7). This sophisticated system forms a weak physical network ex situ through P/C7 peptide binding and in situ network reinforcement through the thermo-induced hydrophobic association of PNIPAM, forming additional junctions [43,44]. As has been shown, soft, medium, and stiff SHIELD variants all resulted in statistically higher levels of cell protection compared to cell delivery in saline. Importantly, the presence or absence of cell-adhesive domain within the SHIELD material did not affect the cell membrane protection. Thus, the shear-thinning/self-healing properties of the gel (and not the gel cell-adhesive properties) are responsible for the cell membrane protection [43]. It seems that the highly tunable one-component simple system (graft copolymer) presented herein can meet the required thermomechanical properties of SHIELD, i.e., two-step gelling (ex situ soft gel, in situ network reinforcement), shear-thinning injectability and instantaneous self-healing, combined with thermo-induced gel stiffening, used for the successful delivery of transplanted stem cells [45].

Shear-thinning alginate hydrogels, crosslinked through ionic interactions in the presence of Ca^2+^ divalent cations, were first identified as material carriers to protect cells from mechanical damage during injection [39]. Provided that the gelator suggested herein is an alginate-based graft copolymer, it is worthy to compare them. As reported, the alginate formulation that produced a hydrogel with *G’* ~30 Pa provided the most effective cell protection for all the cell types investigated. Either increasing or decreasing, the hydrogel storage modulus reduced this protective effect [39]. In the present system, *G’* = 35 Pa for the 4 wt % P(NIPAM_80_-co-NtBAM_20_) in DMEM at 20 °C (Figure 11), which is very close to the value reported for the best cell viability after injection.

As far as the biocompatibility of the NaALG-g-P(NIPAM-co-NtBAM) is concerned, there are some previous reports dealing with analogous grafted polysaccharide-based materials. Particularly, no cytotoxicity or acute systemic toxicity was associated with hyaluronan derivatives, namely HA-g-P(NIPAAm-co-NtBAAm). This injectable and self-assembling scaffold was also compatible with the production of mesenchymal stem/stromal cells (MSC)-derived chondrocytes [46]. Moreover, viability studies with human corneal epithelial (HCE) cells, performed on nanogels of methylcellulose hydrophobized with N-tert-butylacrylamide, demonstrated in vitro biocompatibility [47]. We should finally mention the in vitro and in vivo biocompatibility studies of alginate/Polyacrylamide IPN gels that showed minimal effects on cells, then pure alginate hydrogels [48]. Although these reports are promising, biocompatibility studies are necessary for the polymers under investigation, and this will be an objective of our ongoing research.

## 3. Materials and Methods

### 3.1. Materials

The monomers *N*-isopropylacrylamide (NIPAM) and *N*-tert-butylacrylamide (NtBAM) were used as acquired by Fluorochem (Derbyshire, UK) and Alfa Aesar (Ward Hill, MA, USA), respectively. Potassium peroxodisulfate (KPS, Fluorochem, Derbyshire, UK), 2-aminoethanethiol hydrochloride (ET HCl, Alfa Aesar, Ward Hill, MA, USA), *N*-(3-Dimethylaminopropyl)-*N*′-ethylcarbodiimide hydrochloride (EDC, Alfa Aesar, Ward Hill, MA, USA) and 1-Hydroxybenzotriazole hydrate (HOBt, Fluka, NC, USA) were used as received. Dimethylformamide (DMF, Aldrich, St. Louis, MO, USA), hydrochloric acid (HCl, Panreac, IL, USA), sodium hydroxide (NaOH, Panreac, IL, USA), deuterated water (D_2_O, Sigma-Aldrich, St. Louis, MO, USA) and Dulbecco’s modified Eagle’s medium (DMEM, Sigma-Aldrich, St. Louis, MO, USA) were used as obtained by the provider without purification. Ultrapure water was received by an ELGA Medica-R7/15 device (ELGA Labwater, IL, USA).

Sodium alginate (NaALG, Sigma-Aldrich, St. Louis, MO, USA, no. 180947, molecular weight range: 120,000–190,000 g/mol, the ratio of mannuronic and guluronic units (M/G): 1.53) was further purified (a solution of 7% *w/v* NaALG in NaOH (0.005 M) was repeatedly purified by dialysis against ultrapure water (membrane MWCO: 12,000–14,000 Da), and the final NaALG product was obtained in its solid-state through lyophilization.

### 3.2. Synthesis of the Graft Copolymers

Carbodiimide chemistry was applied for the synthesis of the NaALG based graft copolymers using EDC as condensation agent and HOBt as coupling agent [22,49,50,51]. The experimental procedure concerning the synthesis of the grafting chains is presented in the Supplementary Materials. A typical synthesis of a NaALG-g-PNIPAM graft copolymer was conducted as follows: Primarily, 4 g (0.02 mol of repeat units) of NaALG and 4 g (0.18 mmol) PNIPAM-NH_2_ were separately dissolved in 80 mL ultrapure water each and left under stirring at 23 °C for 24 h. After 24 h, the two aqueous solutions were mixed and left to stir at 23 °C overnight. After full homogeneity, the pH of the solution was adjusted at pH ~ 6 by NaOH (1 M). Next, 0.12 g (0.9 mmol) HOBt was added to the mixture, followed by the addition of 0.67 g (0.0035 mol) EDC. The solution was left under stirring at 23 °C for three days. The graft copolymer was first precipitated in acetone and then redissolved in ultrapure water, while the pH of the final aqueous solution was elevated at pH > 12, adding NaOH (1 M). Finally, the reaction product was received after dialysis against ultrapure water (membrane MWCO: 25,000 Da) and lyophilization. Four different graft copolymers were received through this method, enduing the same procedure by altering the feed composition.

### 3.3. Polymer Characterization

#### 3.3.1. Proton Nuclear Magnetic Resonance, ^1^H NMR

^1^H NMR spectra of NaALG, grafting chains, and NaALG-graft copolymers in D_2_O were obtained using a BRUKER AVANCE III HD PRODIGY ASCEND TM 600 MHz spectrometers (Billerica, MA, USA). NaALG and PNIPAM-NH_2_ were studied at room temperature. The amino-terminated grafting chains P(NIPAM_94_-co-NtBAM_6_)-NH_2_ and the graft copolymer NaALG-g-P(NIPAM_94_-*co*-NtBAM_6_) were studied at 20 °C. The side chains P(NIPAM_86_-*co*-NtBAM_14_)-NH_2_ and P(NIPAM_80_-*co*-NtBAM_20_)-NH_2_ along with the graft copolymers NaALG-g-P(NIPAM_86_-*co*-NtBAM_14_) and NaALG-g-P(NIPAM_80_-*co*-NtBAM)_20_ were studied at 15 °C (details are presented in the Supplementary Materials).

#### 3.3.2. Potentiometric Titration

Acid–base titration of the -NH_2_ end groups was performed to estimate the number average molar mass of the thermo-responsive amino-functionalized grafting chains. Briefly, 0.25 g of the polymer was dissolved in 10 mL ultrapure water. Aiming to fully deprotonated -NH_2_ end-groups, an appropriate amount of NaOH (0.1 M) was added in the polymer solution (pH > 11 was achieved). HCl (0.01 M) was used as the titrant.

#### 3.3.3. Cloud Point Measurements

The phase transition of 0.5% *w/v* aqueous solution of the side chains was observed by turbidimetry at 500 nm using a HITACHI U2001 spectrometer (Illinois/USA). The cloud point, *T_cp_*, was defined as the temperature above, which the optical density starts to increase promptly.

### 3.4. Hydrogels Preparation

Aqueous solutions of NaALG graft copolymers at a concentration of 5 wt % were prepared and left under stirring at 20 °C on a lab shaker (200 rpm) equipped with a refrigerated bath circulator. After homogeneity, the pH of the solutions was tuned at pH 7.4, using NaOH (1 M).

### 3.5. Rheological Studies

A stress-controlled AR-2000ex (TA Instruments, New Castle, DE, USA) rheometer with a cone and plate geometry (diameter 20 mm, angle 3°, truncation 111 μm) was used for the rheological study of the NaALG graft copolymer aqueous solutions. The experiments were performed in the linear viscoelastic regime, which was determined by strain sweep tests at a frequency of 1 Hz. The temperature was controlled by a Peltier system with an accuracy of ± 0.1 °C. The rheometer was equipped with a solvent trap to avoid concentration changes due to water evaporation.

## 4. Conclusions

A series of graft copolymers (with relatively low grafting density) consisted of alginate backbone and P(NIPAM_x_-*co*-NtBAM_y_) grafting chains were synthesized and explored as gelators in various media. All the NaALG-*g*-P(NIPAM_x_-*co*-NtBAM_y_) copolymers, at a concentration of 5 wt %, exhibit thermo-induced gelation in aqueous media upon heating, owing to the hydrophobic intermolecular association of their pendant grafting chains. The enrichment of PNIPAM with the hydrophobic NtBAM monomer units resulted in a linear decline of their cloud points with NtBAM content. This induced a considerable shift of the gelation point, *T_gel_*, to lower temperatures, shifting the gelation window accordingly. *T_gel_* was lowered from 38 to 20 °C by varying the NtBAM content from 0 to 20 mol %. The linear dependence of *T_gel_* with the NtBAM content of the grafting chains seems to be the determining factor of *T_gel_,* at least at low grafting density, which allows fine-tuning of the gelation temperature window. The impact of the *T_gel_* shift on the rheological properties of the formed hydrogels is significant at room and body temperature. For instance, at 37 °C, the storage modulus increases about two orders of magnitude and the terminal relaxation time increase about 10 orders of magnitude by enriching the sticky grafting chains with 20% mol hydrophobic moieties.

The overall temperature-dependent rheological properties of the graft copolymers reveal two main behaviors, a sol–gel and a weak gel-stiff gel transition for the copolymer with stickers of low (0, 6% mol) and high (14, 20% mol) NtBAM content, respectively. The first type of hydrogels is evidently injectable since they flow easily at room temperature. For the second type of hydrogels, their injectability is based on their additional shear thinning behavior. To prove this, stepwise shear-dependent experiments, designed to simulate injection through a 28-gauge needle syringe, were conducted for the 5 wt % NaALG-*g*-P(NIPAM_80_-*co*-NtBAM_20_)/water formulation. The results showed excellent shear-induced responsiveness. More importantly, the combination of thermo- and shear-responsiveness provides excellent injectability.

To examine its potential use in bioapplications, the NaALG-*g*-P(NIPAM_80_-*co*-NtBAM_20_) copolymer was explored as a gelator in various environments of biological interest. The various constituents of the media, especially salts, affect the rheological properties of the formulations due to their interaction with both alginate backbone and PNIPAM-based stickers. These effects must be taken into account to tailor the rheological behavior of the hydrogels. When a cell cultivating media (e.g., DMEM) was used, the rheological properties differ significantly concerning those in the other media. The 4 wt % NaALG-*g*-P(NIPAM_80_-*co*-NtBAM_20_)/DMEM formulation exhibits excellent shear-induced injectability and instantaneous thermo-induced gel stiffening at physiological temperature. The rheological properties (e.g., storage modulus at room and body temperature, shear-thinning, self-healing) of this formulation resemble those explored for cell transplantation using shear-thinning/self-healing hydrogels (e.g., SHIELD) as carriers. Considering the biocompatibility of PNIPAM-based grafted polysaccharides [46,47,52,53,54], this formulation seems a good candidate for further investigation, targeting cell transplantation potential applications.

## Figures and Tables

**Table 1:** Molecular characteristics of the grafting chains.

Polymer	M_n_^a^ (g/mol)	Theoretical NIPAM/NtBAM Molar Ratio	NIPAM/NtBAM Molar Ratio ^b^	T*_cp_* ^c^(°C)
PNIPAM-NH_2_	22,700	100/0	100/0	32
P(NIPAM_94_-*co*-NtBAM_6_)-NH_2_	14,800	95/5	94/6	30
P(NIPAM_86_-*co*-NtBAM_14_)-NH_2_	17,000	85/15	86/14	22
P(NIPAM_80_-*co*-NtBAM_20_)-NH_2_	16,900	80/20	80/20	20

^a^ from acid–base titration; ^b^ from proton nuclear magnetic resonance (^1^H NMR); ^c^ from turbidimetry, defined at the onset of the optical density abrupt increase.

**Table 2:** Molecular characteristics of the graft copolymers from ^1^H NMR.

Polymer	Mw (×10^3^ g/mol) ^a^	% Weight Composition Alg/Grafting Chains (wt/wt)	Grafting ^b^ Density
NaALG-*g*-PNIPAM	203	69/31	3
NaALG-*g*-P(NIPAM_94_-*co*-NtBAM_6_)	280	50/50	8
NaALG-*g*-P(NIPAM_86_-*co*-NtBAM_14_)	222	63/37	5
NaALG-*g*-P(NIPAM_80_-*co*-NtBAM_20_)	179	78/22	3

^a^ Calculated from the alginate Mw = 140,000 g/mol and its% weight composition from ^1^H NMR. ^b^ number of grafting chains per alginate backbone by ^1^H NMR.

## Data Availability

Not applicable.

## Supplementary Materials

The following are available online at https://www.mdpi.com/article/10.3390/ijms22083824/s1, Table S1, Synthesis conditions of the grafting chains; Figure S1, ^1^H-NMR spectrum of PNIPAM-NH2; Figure S2, ^1^H-NMR spectrum of P(NIPAM94-co-NtBAM6)-NH2; Figure S3, ^1^H-NMR spectrum of P(NIPAM86-co-NtBAM14)-NH2; Figure S4, ^1^H-NMR spectrum of P(NIPAM80-co-NtBAM20)-NH2; Figure S5, ^1^H-NMR spectra of left: NaALG-g-PNIPAM; right: NaALG; Figure S6, ^1^H-NMR spectra of: NaALG-g-P(NIPAM80-co-NtBAM20), NaALG-g-P(NIPAM86-co-NtBAM14), NaALG-g-P(NIPAM94-co-NtBAM6); Figure S7, G’ (closed), G’’ (open) versus temperature (cooling/heating ramp) at 1 Hz, strain amplitude of 0.1 % and Cp=5 wt % for different graft copolymers: NaALG-g-ΡΝΙΡΑΜ, NaALG-Ρ(ΝΙΡΑΜ94-co-NtBAM6), NaALG-Ρ(ΝΙΡΑΜ86-co-NtBAM14); Figure S8, ΔΤ = Tgel, heating -Tgel, cooling versus ramp rate (°C/min) for the NaALG-g-PNIPAM (Cp =5 wt %, strain 0.5 %, at 1 Hz); Figure S9, Frequency dependence of tanδ for the NaALG-g-P(NIPAMx-co-NtBAMy)/water systems of different monomer composition in the grafting chains, as indicated (inset), at 37 °C; Figure S10, Frequency dependence of G’, G’’ for NaALG-g-P(NIPAM80-co-NtBAM20) Cp = 5 wt%) at γ=0.1% and at various temperatures; Figure S11, Frequency dependence of G’ (closed), G’’ (open) for NaALG-g-Ρ(ΝΙΡΑΜ86-co-NtBAM14) (left) and NaALG-g-P(NIPAM80-co-NtBAM20) (right) at γ=0.1% and Cp = 5 wt%; Figure S12, Shear viscosity as a function of shear rate (decreasing from 100 s-1) at different temperatures, as indicated; Figure S13, Temperature dependence of G’ (closed), G’’ (open) (cooling/heating ramp, 1 °C/min) at 1 Hz and strain amplitude of 0.1 % for the NaALG-g-P(NIPAM80-co-NtBAM20) graft copolymer (Cp=5 wt %) in various media; Table S2, Tgel and G’ (37 °C) of 5 wt% NaAlg-g-P(NIPAM80-co-NtBAM20) in various media; Figure S14, Storage modulus G’ (squares, black) and loss modulus G’’ (circles, red) versus strain at 20 °C followed by time sweep, immediately after switching temperature and strain at 37 °C and 0.1%.
