# Supplementary material for: Alginate-g-PNIPAM-Based Thermo/Shear-Responsive Injectable Hydrogels: Tailoring the Rheological Properties by Adjusting the LCST of the Grafting Chains
Source: Int J Mol Sci. 2021 Apr 7;22(8):3824. doi: 10.3390/ijms22083824 (PMC8067843; doi:10.3390/ijms22083824)
Supplement: Supplementary file 1 [file ijms-22-03824-s001.pdf]

Supporting Information

Alginate-*g*-PNIPAM-based Thermo/shear-responsive Injectable Hydrogels: Tailoring the Rheological Properties by Adjusting the LCST of the Grafting Chains

Konstantinos Safakas[1], Sofia-Falia Saravanou[1], Zacharoula Iatridi[1], Constantinos Tsitsilianis[1,*]

1. Department of Chemical Engineering, University of Patras, 26500 Patras, Greece; kostassaf@hotmail.com (K.S); faliasaravanou@hotmail.com (S-F.S.); iatridi@upatras.gr (Z.I)
*Correspondence: Prof. Constantinos Tsitsilianis; Department of Chemical Engineering, University of Patras, 26500 Patras, Greece; Phone: +30 2610 969531; E-mail address: ct@chemeng.upatras.gr

Synthesis of homopolymers PNIPAM-NH_2

Initially, 20 g of NIPAM (0.177 mol) were dissolved in ultrapure water (the concentration of NIPAM was 1M) and the aqueous solution was degassed with nitrogen bubbling for 40 minutes. Afterwards, proper quantities of KPS (1% moles over the NIPAM moles) and AET HCl (0.5% moles over the NIPAM moles) were dissolved in 5 mL of ultrapure water, independently and added in the NIPAM solution. The final mixture was degassed with nitrogen for 4 h and the reaction temperature was set at 29 ^{o}C for 24 h. The final product was purified from impurities through dialysis against pure water (membrane MWCO: 12000–14000 Da) and was received in solid state by freeze drying (**Table S1**).

Synthesis of random copolymers P(NIPAM$_x$-co-NtBAM$_y$)-NH_2

The amino-functionalized P(NIPAM$_x$-*co*-NtBAM$_y$)-NH_2 grafting chains were synthesized through radical polymerization in presence of KPS as initiator and AET HCl as chain transfer agent.[1,2] For the synthesis of P(NIPAM$_{94}$-co-NtBAM$_6$)-NH_2 , 0.24 g (0.0019 mol) NtBAM were dissolved in DMF. Adequate amount of ultrapure water was added in the mixture until no phase separation was observed. Next, 4g (0.0354 mol) NIPAM were added in the solution. The monomers concentration in the mixture of solvents was 1M. The mixture was degassed with nitrogen bubbling for 40 minutes. At a next step, proper amounts of KPS (2% moles over the total monomers moles) and AET HCl (1% moles over the total monomers moles) were dissolved individually in 2 mL of ultrapure water and then were added in the solution. The final mixture was degassed with nitrogen for 4 h and the reaction temperature was set at 29 °C for 24 h. Analogous method was followed for the synthesis of two more random copolymers grafting chains. The reaction conditions for all the homopolymer and copolymer grafting chains are tabulated in Table S1.

Table S1. Synthesis conditions of the grafting chains.

Grafting chains	NIPAM (g)	NtBAM (g)	KPS (% mol)	AET HCl (% mol)
PNIPAM-NH_2	20	0	1 [a]	0.5 [a]
P(NIPAM$_{94}$-*co*-NtBAM$_6$)-NH_2	4	0.24	2 [b]	1 [b]
P(NIPAM$_{86}$-*co*-NtBAM$_{14}$)-NH_2	15	2.98	1 [b]	0.5 [b]
P(NIPAM$_{80}$-*co*-NtBAM$_{20}$)-NH_2	16	4.50	1 [b]	0.5 [b]

a. Relative to the moles of NIPAM.

b. Relative to the moles of NIPAM and NtBAM.

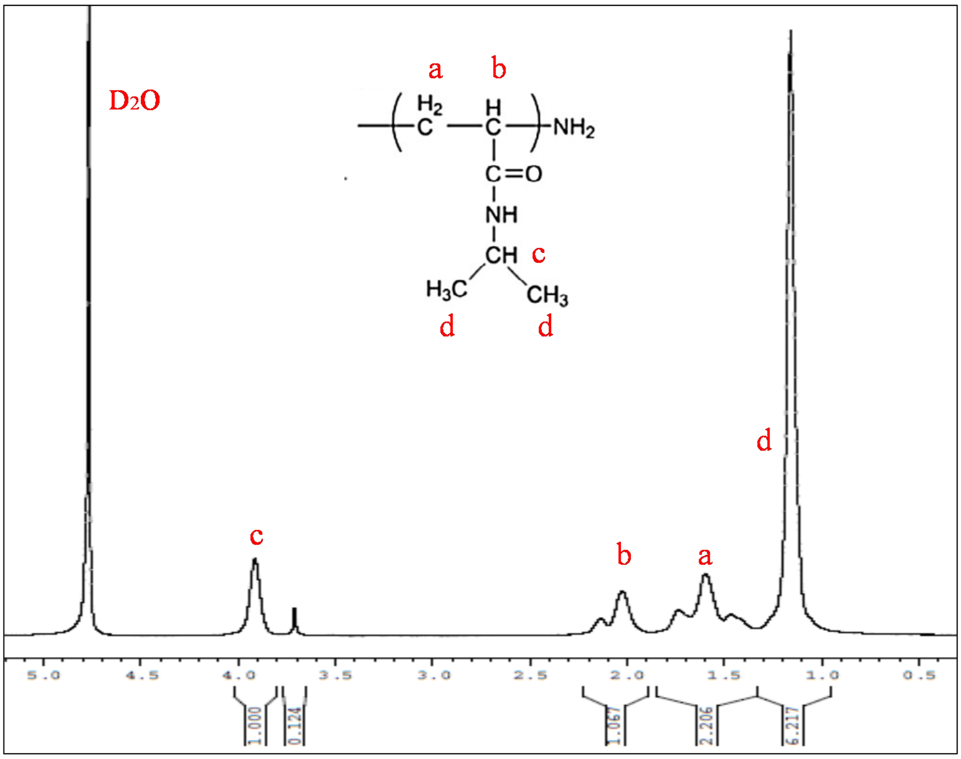

Figure S1. ^{1}H-NMR spectrum of PNIPAM-NH_2.

The successful synthesis of the PNIPAM-NH_2 homopolymer was verified in the ^{1}H-NMR spectrum presented in **Figure S1**. The characteristic peak of the solvent used (deuterated water, D_2O) at 4.8ppm can be observed. There also can be seen the characteristic peaks of the protons of the methylene and methine groups (1.3-2.2 ppm), the six methyl protons of the isopropyl group (-CH_3) (~1.1 ppm) and the one proton of the isopropyl group linked to the amide group (N-C-H) (~3.9 ppm) of NIPAM.

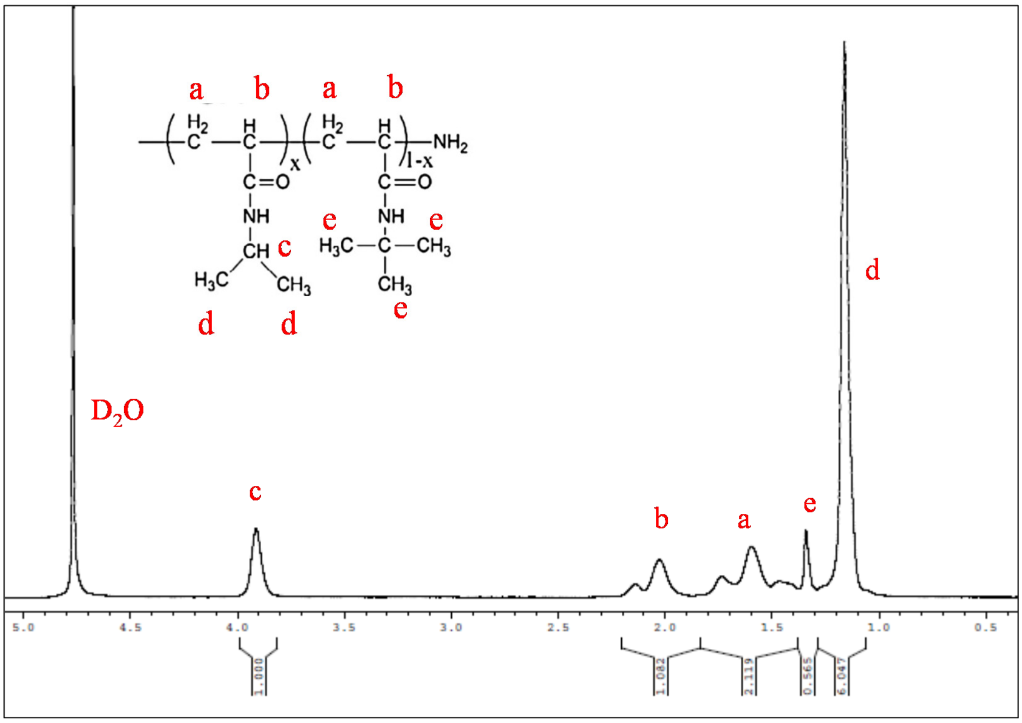

Figure S2. ^{1}H-NMR spectrum of P(NIPAM$_{94}$-*co*-NtBAM$_{6}$)-NH$_{2}$.

The ^{1}H-NMR spectra of the three copolymers P(NIPAM$_{94}$-co-NtBAM$_{6}$)-NH$_{2}$, P(NIPAM$_{86}$-co-NtBAM$_{14}$)-NH$_{2}$ and P(NIPAM$_{80}$-*co*-NtBAM$_{20}$)-NH$_{2}$ are shown in **Figures S2, S3** and **S4**, respectively. Here, again, the characteristic peak of D_2O at 4.8ppm is detected. Also, the peaks at 1.3-2.2 ppm represent the methylene and methine protons of NIPAM and NtBAM, the one proton of the isopropyl group linked to the amide group (N-C-H) of NIPAM at 3.9 ppm, the six methyl protons of the isopropyl group (-CH$_{3}$) of NIPAM at 1.1 ppm, and the nine methyl protons of the –CH$_{3}$ groups of NtBAM at 1.2 ppm.[1,2,3] The NIPAM and NtBAM monomers content of the copolymers was calculated comparing and integrating the areas of the peaks at 1.2 ppm (9 protons of the -CH$_{3}$ groups of NtBAM) and 3.9 ppm (1 proton of the N-C-H group of NIPAM).[1,2,4]

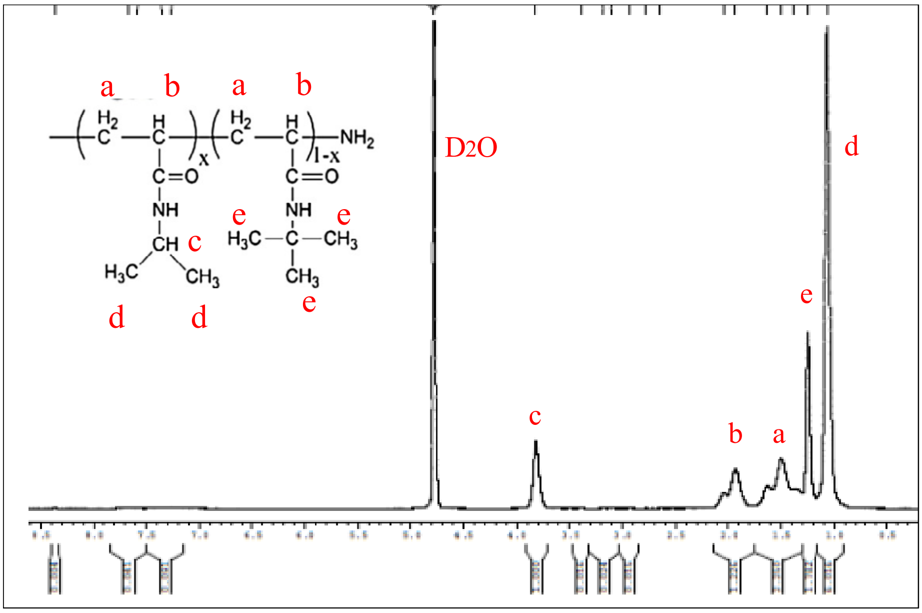

Figure S3. ^{1}H-NMR spectrum of P(NIPAM$_{86}$-*co*-NtBAM$_{14}$)-NH$_2$.

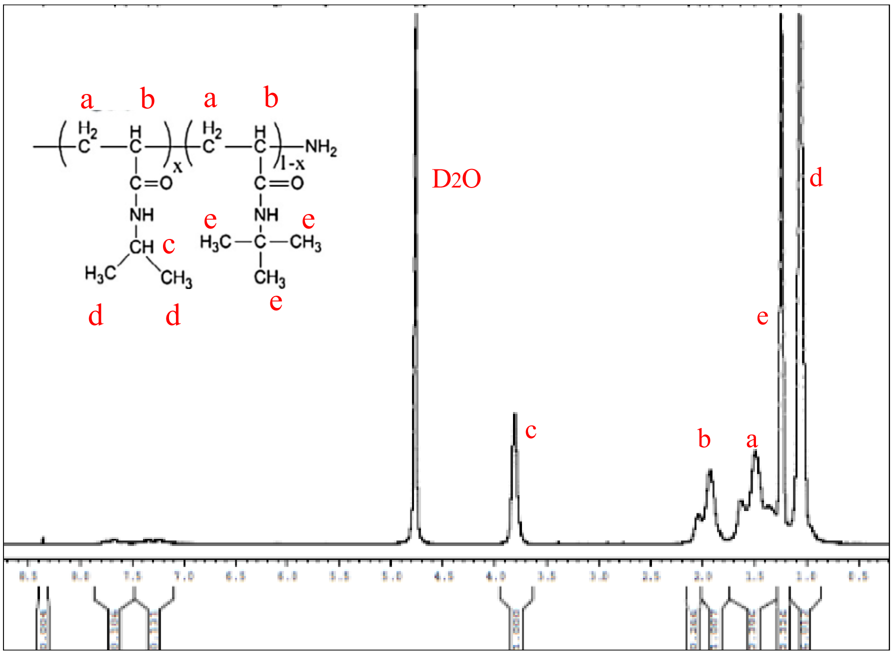

Figure S4. ^{1}H-NMR spectrum of P(NIPAM$_{80}$-*co*-NtBAM$_{20}$)-NH$_2$.

In the ^{1}H NMR spectrum of the graft copolymer NaALG-g-PNIPAM (**Figure S5 left**), all the characteristic peaks of the protons of PNIPAM (as described in Figure S1) are present. In the **right Figure S5**, where the spectrum of pure alginate is given for reasons of comparison, one can observe the peaks at 3.5-4.6 ppm that correspond to four protons of alginate.[5] These peaks, overlapped by the one proton of the isopropyl group linked to the amide group (N-C-H) of NIPAM at 3.9 ppm, also exist in the spectrum of the NaALG-g-PNIPAM copolymer, confirming the successful grafting of the thermo-responsive chains onto alginate. From the integration of the peaks at 3.5-4.6 ppm (4 protons of alginate ring and 1 proton of PNIPAM) and the peaks at 1.0-2.2 ppm (corresponding to characteristic peaks of the protons of the methylene and methine groups and the six methyl protons of the isopropyl group ($-CH_3$) of PNIPAM), the weight composition (w/w) and the grafting density (grafting chains/alginate, mol/mol) of the graft copolymers were found.

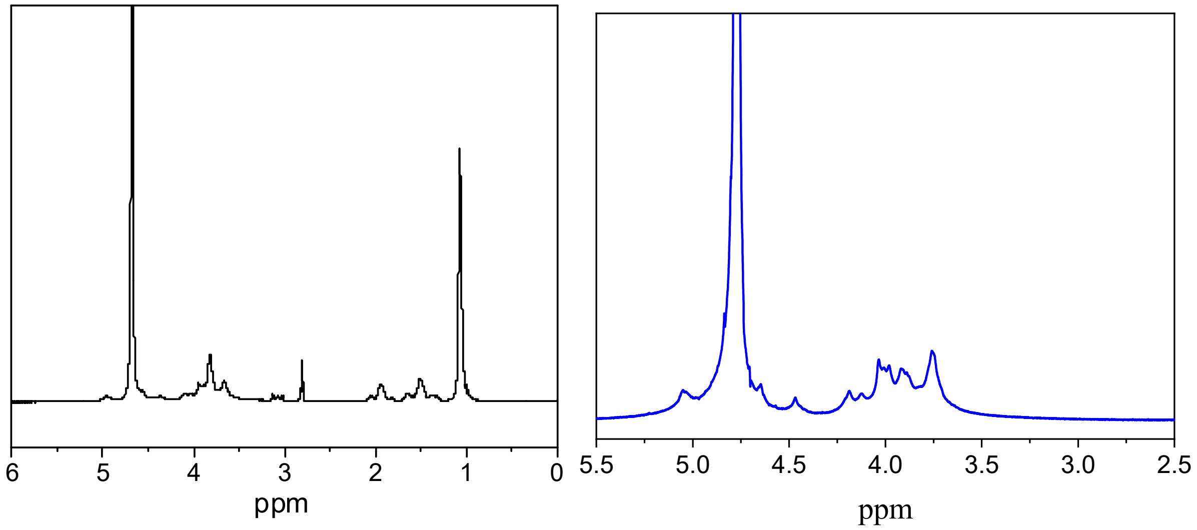

Figure S5. ^{1}H-NMR spectra of left: NaALG-g-PNIPAM; right: NaALG.

Likewise, all the characteristic peaks of the alginate backbone as well as the grafting chains P(NIPAM$_x$-*co*-NtBAM$_y$) were present in the spectra of the alginate-based graft copolymers NaALG-g-P(NIPAM$_{94}$-*co*-NtBAM$_6$), NaALG-g-P(NIPAM$_{86}$-*co*-NtBAM$_{14}$) and NaALG-g-P(NIPAM$_{80}$-*co*-NtBAM$_{20}$) (**Figure S6**). Here, the weight composition (w/w) and the grafting density of the graft copolymers were calculated comparing the integrated areas at 3.5-4.6 ppm (4 protons of alginate ring and 1 proton of P(NIPAM$_x$-*co*-NtBAM$_y$)) and at 1.0-

2.2 ppm (corresponding to characteristic peaks of the protons of the P(NIPAM$_x$-*co*-NtBAM$_y$) grafting chains).

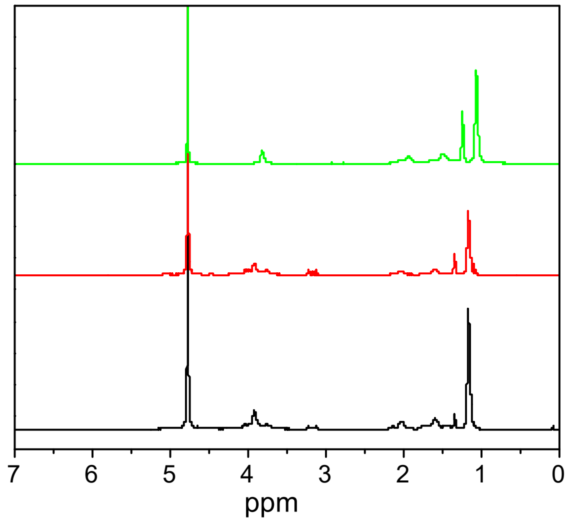

Figure S6. ^{1}H-NMR spectra from top to bottom: NaALG-g-P(NIPAM$_{80}$-*co*-NtBAM$_{20}$), NaALG-g-P(NIPAM$_{86}$-*co*-NtBAM$_{14}$), NaALG-g-P(NIPAM$_{94}$-*co*-NtBAM$_6$).

Additional rheological data

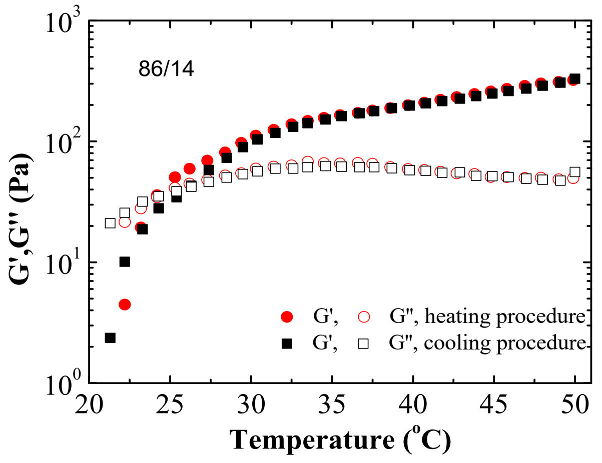
86/14
G', G", heating procedure
G', G", cooling procedure
G',G" (Pa)
Temperature (oC)

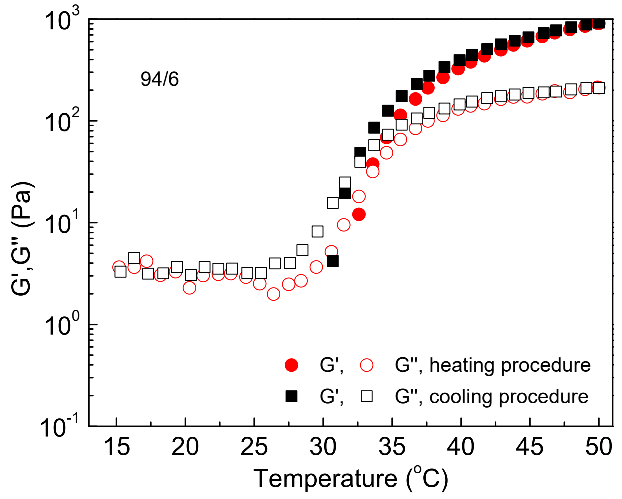
94/6
G', G", heating procedure
G', G", cooling procedure
G',G" (Pa)
Temperature (oC)

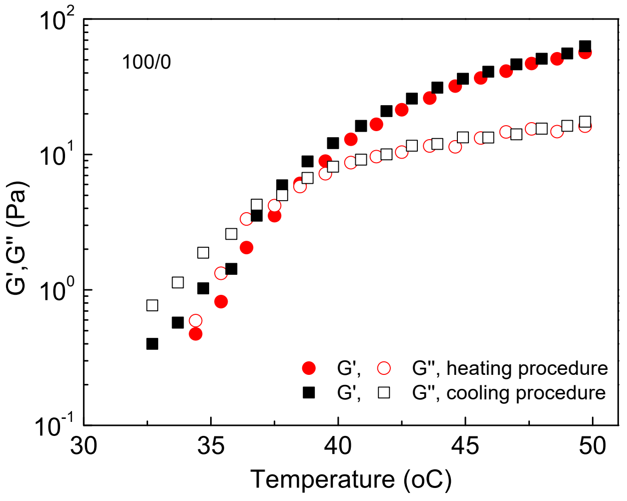
100/0
G', G", heating procedure
G', G", cooling procedure
G',G" (Pa)
Temperature (oC)

Figure S7. G' (closed), G'' (open) versus temperature (cooling/heating ramp) at 1 Hz, strain amplitude of 0.1 % and C_p=5 wt % for different graft copolymers: NaALG-g-PNIPAM, NaALG-P($NIPAM_{94}$-*co*-$NtBAM_6$), NaALG-P($NIPAM_{86}$-*co*-$NtBAM_{14}$).

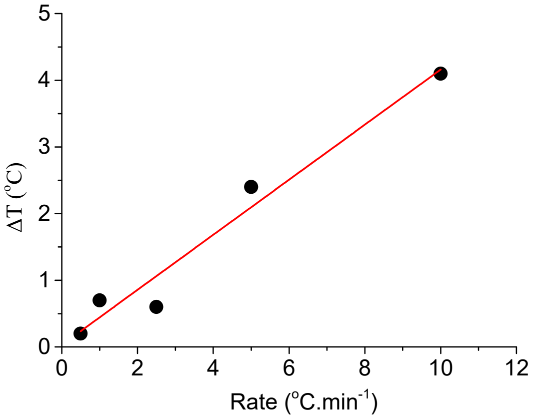

Figure S8. $\Delta T = T_{gel,\ heating} - T_{gel,\ cooling}$ versus ramp rate (°C/min) for the NaALG-g-PNIPAM (C_p =5 wt %, strain 0.5 %, at 1 Hz).

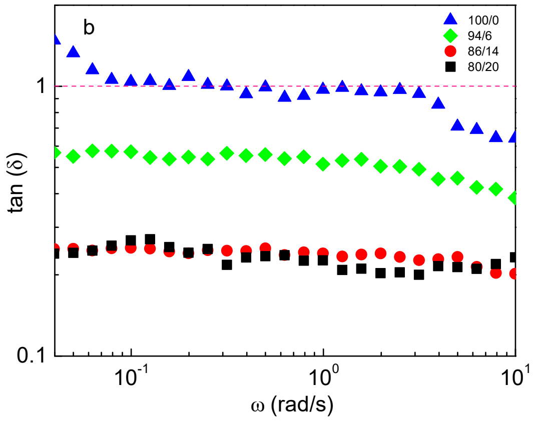

Figure S9. Frequency dependence of tanδ for the NaALG-g-P($NIPAM_x$-*co*-$NtBAM_y$)/water systems of different monomer composition in the grafting chains, as indicated (inset), at 37 °C.

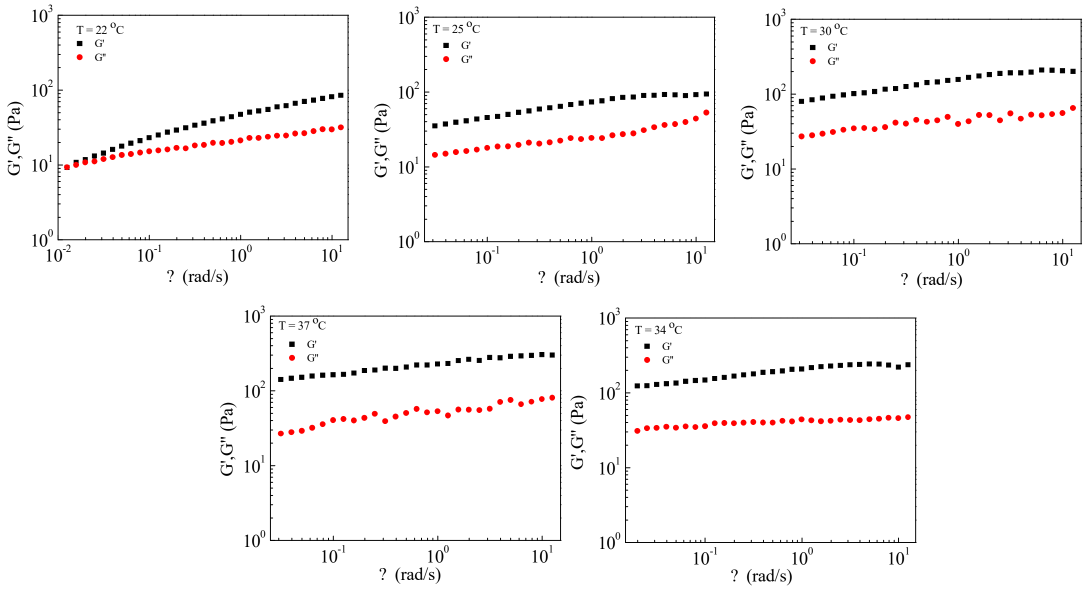

Figure S10. Frequency dependence of G', G'' for NaALG-g-P(NIPAM$_{80}$-*co*-NtBAM$_{20}$) C_p=5 wt%) at γ=0.1% and at various temperatures.

Comparison of activation energy

We could compare the value estimated here in (E_a=870 kJ/mol) with that estimated by Onoda et al.[6] by using the proportionality $E_a \sim N^{2/3}\gamma$, where N is the degree of polymerization of the sticky chains and their surface tension with the solvent. For the stickers P(NIPAM$_{80}$-*co*-NtBAM$_{20}$) of our polymer N_A=146 and for the P(NIPAM$_{82}$-*co*-butyl acrylate$_{18}$) N_B=89. Thus, in our case E_a should be higher by a factor of $(N_A/N_B)^{2/3}$.

Hence: $(146/89)^{0.666}$=1.39 and E_a=1.39 x 619 kJ/mol =860 kJ/mol, slightly lower than the estimated value, which likely is due to the slightly different γ.

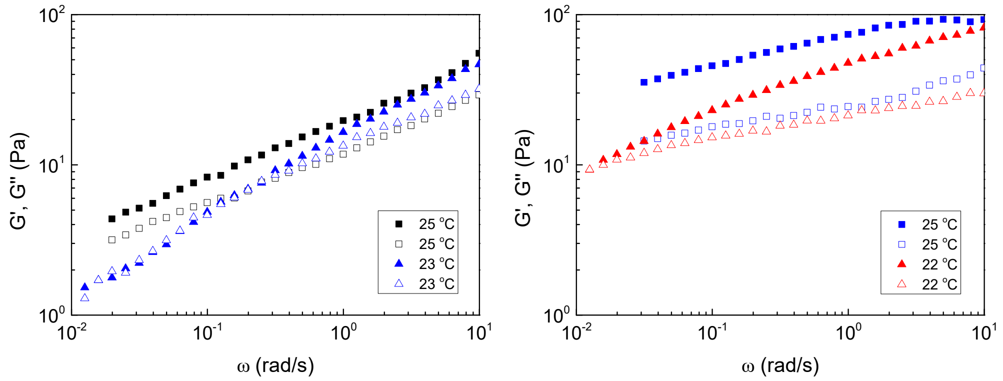

Figure S11. Frequency dependence of G' (closed), G'' (open) for NaALG-g-P(NIPAM$_{86}$-*co*-NtBAM$_{14}$) (left) and NaALG-g-P(NIPAM$_{80}$-*co*-NtBAM$_{20}$) (right) at γ=0.1% and C_p=5 wt%.

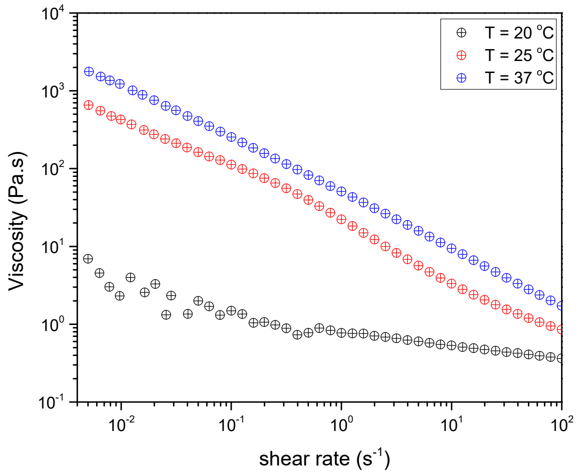

Figure S12. Shear viscosity as a function of shear rate (decreasing from 100 s^{-1}) at different temperatures, as indicated.

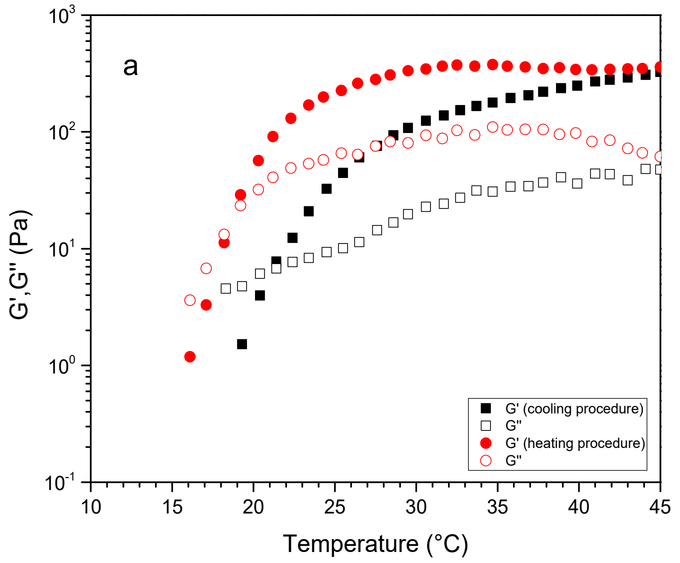
a
G',G'' (Pa)
Temperature (°C)
G' (cooling procedure)
G''
G' (heating procedure)
G''

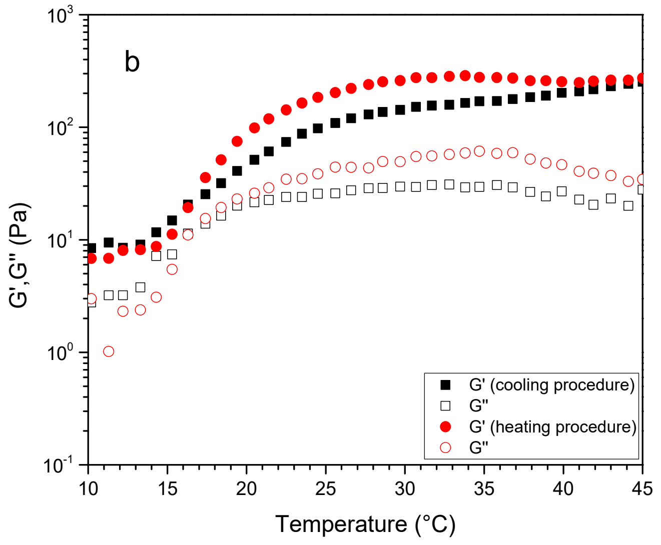
b
G',G'' (Pa)
Temperature (°C)
G' (cooling procedure)
G''
G' (heating procedure)
G''

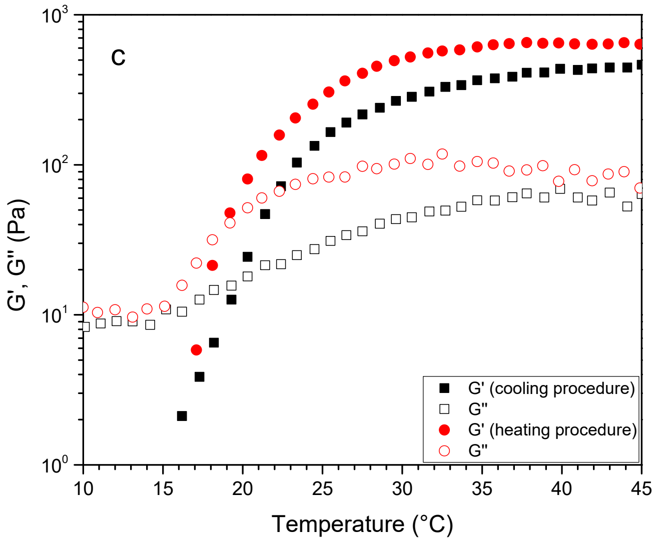

Figure S13. Temperature dependence of G’ (closed), G’’ (open) (cooling/heating ramp, 1 °C/min) at 1 Hz and strain amplitude of 0.1 % for the NaALG-g-P(NIPAM$_{80}$-*co*-NtBAM$_{20}$) graft copolymer (C_p=5 wt %) in various media (a), PB, (b) PBS, (c) DMEM.

Table S2. T_{gel} and *G’* (37 °C) of 5 wt% NaAlg-g-P(NIPAM$_{80}$-*co*-NtBAM$_{20}$) in various media.

solution	T_{gel} (°C)	G’ (Pa) / 37 °C
H_2O	19.8	266.5
PB (1mM)	18.6	359.5
PBS (1mM, 0.135M NaCl)	n. o.	273.5
DMEM	18.9	642.3
DMEM/ C_p 4 wt%	17.6	260.1

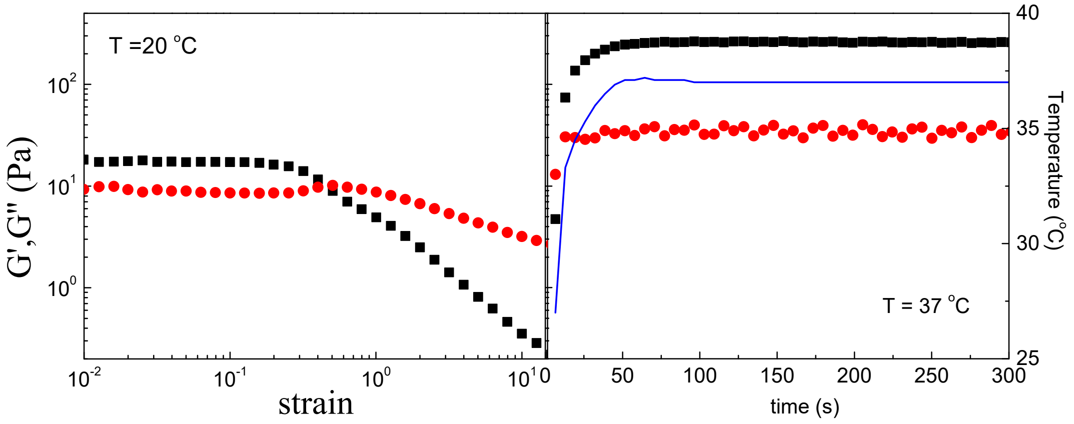

Figure S14. Storage modulus G' (squares, black) and loss modulus G'' (circles, red) versus strain at 20 °C followed by time sweep, immediately after switching temperature and strain at 37 °C and 0.1% respectively, of a 4 wt% NaALG-g-P(NIPAM$_{80}$-*co*-NtBAM$_{20}$) in DMEM at pH 7.4. The blue line represents the evolution of temperature from 20 to 37 °C as captured by the rheometer.

(1) Soledad Lencina, M. M.; Iatridi, Z.; Villar, M. A.; Tsitsilianis, C. Thermoresponsive hydrogels from alginate-based graft copolymers. *Eur. Polym. J.* **2014**, *61*, 33–44.

(2) Iatridi, Z.; Saravanou, S.-F.; Tsitsilianis, C. Injectable self-assembling hydrogel from alginate grafted by p(N-isopropylacrylamide-co-N-tert-butylacrylamide) random copolymers. *Carbohydr. Polym.* **2019**, *219*, 344-352.

(3) Rwei, S.-P.; Chuang, Y.-Y.; Way, T.-F.; Chiang, W.-Y. Thermosensitive copolymer synthesized by controlled living radical polymerization: Phase behavior of diblock copolymers of poly(N-isopropyl acrylamide) families. *J. Appl. Polym. Sci.* **2016**, *133*, 43224.

(4) Gibbons, O.; Carroll, W. M.; Aldabbagh, F.; Yamada, B. Nitroxide-mediated controlled statistical copolymerizations of N-isopropylacrylamide with N-tert-butylacrylamide. *J. Polym. Sci., A: Polym. Chem.* **2006**, *44*, 6410–6418.

(5) Guo, H.; de Magalhaes Goncalves, M.; Ducouret, G.; Hourdet, D. Cold and Hot Gelling of Alginate-graft-PNIPAM: a Schizophrenic Behavior Induced by Potassium Salts. *Biomacromolecules* **2018**, *12, 19(2)*, 576-587.

(6) Onoda, M.; Ueki, T.; Tamate, R.; Akimoto, A. M.; Hall, C. C.; Lodge, T. P.; Yoshida, R. Precisely Tunable Sol-Gel Transition Temperature by Blending Thermoresponsive ABC Triblock Terpolymers. *ACS Macro Lett.* **2018**, *7*, 950-955.
